# A Resource-Based Modelling Framework to Assess Habitat Suitability for Steppe Birds in Semiarid Mediterranean Agricultural Systems

**DOI:** 10.1371/journal.pone.0092790

**Published:** 2014-03-25

**Authors:** Laura Cardador, Miquel De Cáceres, Gerard Bota, David Giralt, Fabián Casas, Beatriz Arroyo, François Mougeot, Carlos Cantero-Martínez, Judit Moncunill, Simon J. Butler, Lluís Brotons

**Affiliations:** 1 Forest Sciences Center of Catalonia (CTFC), Solsona, Catalonia, Spain; 2 Estación Experimental de Zonas Áridas (EEZA-CSIC), La Cañada de San Urbano, Almería, Spain; 3 Instituto de Investigación en Recursos Cinegéticos (IREC)-(CSIC-UCLM-JCCM), Ciudad Real, Spain; 4 Departament de Producció Vegetal i Ciència Forestal, Universidad de Lleida (UDL), Lleida, Spain; 5 School of Biological Sciences, University of East Anglia, Norwich, United Kingdom; 6 CREAF, Bellaterra, Catalonia, Spain; Institute of Agronomy, University of Lisbon, Portugal

## Abstract

European agriculture is undergoing widespread changes that are likely to have profound impacts on farmland biodiversity. The development of tools that allow an assessment of the potential biodiversity effects of different land-use alternatives before changes occur is fundamental to guiding management decisions. In this study, we develop a resource-based model framework to estimate habitat suitability for target species, according to simple information on species’ key resource requirements (diet, foraging habitat and nesting site), and examine whether it can be used to link land-use and local species’ distribution. We take as a study case four steppe bird species in a lowland area of the north-eastern Iberian Peninsula. We also compare the performance of our resource-based approach to that obtained through habitat-based models relating species’ occurrence and land-cover variables. Further, we use our resource-based approach to predict the effects that change in farming systems can have on farmland bird habitat suitability and compare these predictions with those obtained using the habitat-based models. Habitat suitability estimates generated by our resource-based models performed similarly (and better for one study species) than habitat based-models when predicting current species distribution. Moderate prediction success was achieved for three out of four species considered by resource-based models and for two of four by habitat-based models. Although, there is potential for improving the performance of resource-based models, they provide a structure for using available knowledge of the functional links between agricultural practices, provision of key resources and the response of organisms to predict potential effects of changing land-uses in a variety of context or the impacts of changes such as altered management practices that are not easily incorporated into habitat-based models.

## Introduction

Traditional low-intensity agricultural systems are often associated with high biodiversity conservation value in different regions of the world [1]. As a result of thousands of years of agricultural expansion, a large number of wild species live on land dedicated to human food production, and their preservation strongly depends on traditional low-intensity practices [2], [3]. This is particularly relevant in some regions, such as Europe, where agricultural landscapes represent the major part (about 60%) of non-urban areas [2], [3]. Here, traditional agricultural systems, based on low intensive farming and extensive grazing, have historically provided highly heterogeneous landscapes capable of holding species-rich communities of organisms [4]. However, in recent decades these systems have come under pressure due to socio-economic changes, increased food demands and new technological opportunities [2]. As a result, farmland in many industrialized countries is being profoundly altered, mainly through agricultural intensification and land abandonment, posing a major challenge for biodiversity conservation today [5], [6]. Unless the detrimental impacts of present and future agricultural practices can be prevented or mitigated, many agricultural landscapes will suffer from further degradation in the coming decades [2]. Managing the environmental effects of these agricultural changes thus requires the development of frameworks that allow the exploration of their potential threats and opportunities, even before the changes occur [7]–[9].

Habitat models may provide valuable tools for predicting species’ responses to different land-use alternatives. To date, the potential effects of changing landscapes on species’ dynamics and biodiversity have tended to be addressed through correlative models whereby species-habitat associations are estimated by statistically relating current distributions to particular structural land cover types [10], [11]. When habitat conditions remain fixed temporally and spatially and there is appropriate information to use as a surrogate for factors relevant to species’ habitat selection [12], such habitat association models can be successful at predicting species’ occurrence or population dynamics from habitat characteristics. However, they can be much less successful when used to make predictions outside the area or habitat conditions for which the model has been calibrated [13]. As a consequence, it has recently been proposed that, instead of using structural land cover types, land use – population dynamics relationships might be better examined in the context of functional cover types, such as foraging or nesting habitat, identified on the basis of resource dependencies of species or species’ groups [14], [15].

Unlike habitat-based approaches, resource-based models assess the relative quality of a selected habitat type (e.g. crop or agricultural practice) on the basis of key factors underpinning the distribution and abundance of the considered species [11], [16]. For example, species’ habitat associations, if present, are largely dictated by the availability of key resources in such habitats, rather than the habitat *per se* [14], [15]. Basing distribution and abundance models on resource availability rather than habitat types is therefore likely to allow more robust predictions, even under changing environmental conditions. This is particularly important in farmland landscapes, where intra- and inter-annual changes in crop types and agricultural management are usual [15], [17]. However, to date there has been little application of resource-based models with predictive purposes in conservation studies because they are often difficult to build, relying on robust knowledge of the biology of the target organisms and about the habitat [18].

In this study, we develop a resource-based model framework to estimate habitat suitability for target species, according to simple information on species’ key resource requirements, and examine whether it can be used to link land-use and local species’ distribution. We adopt a simple definition of a species’ requirements, characterised by diet, foraging habitat and nesting site, because previous research has shown significant associations between changes in the expected availability of these resources and population trends [7], [8], [15]. We use this framework to evaluate the ability of different cover types to provide suitable and sufficient resources to support viable populations of farmland bird species during the breeding period, taking as a study case four steppe bird species in a lowland area within north-eastern Iberian Peninsula. We validate our model by determining the relationship between resource-based habitat suitability predictions and information on species’ local distribution. We also compare the performance of our resource-based approach to that of a simple habitat-based model statistically relating species’ occurrence and structural cover types. Finally, we use our approach to predict the effect that changes in farming systems can have on farmland bird habitat suitability, and compare these predictions with those obtained using the habitat-based model.

## Materials and Methods

### Ethics Statement

The project had the permission of the relevant national authorities (i.e., Direcció General de Medi Natural, Departament d’Agricultura, Ramaderia, Pesca, Alimentació i Medi Natural de la Generalitat de Catalunya). Field work conducted was not invasive and did not require the manipulation of live animals. All censuses were performed using public rural tracks and no additional permission to access to privately owned land was needed.

### Study Area and Species

**Figure 1:**
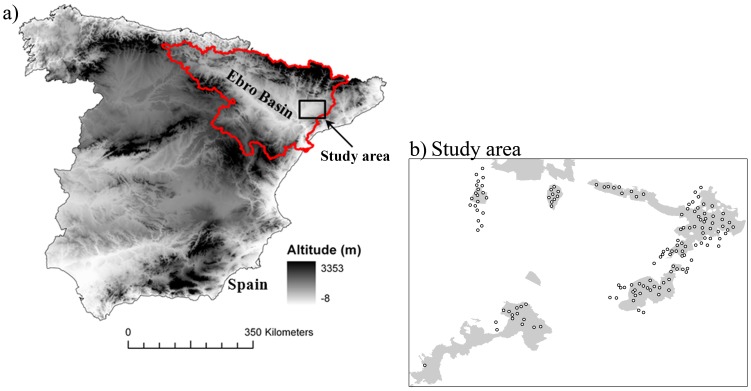
Study area. (a) Location of the study area and (b) distribution of monitored transects (represented by dots) during years 2010–2011 in the study area. In grey, areas included in the Natura 2000 network.

The study area is located in the Catalan Ebro basin, north-eastern Spain (Fig. 1a, b). This area comprises around 400 km^2^ of farmlands, mostly included in Special Protection Areas (SPA), sites established under 2009/147/EC Birds Directive and included in Natura 2000 network (i.e., the European network of nature protection areas). The landscape is predominantly flat and low altitude, broken by discrete ranges of small hills (0–400 m asl), and has a semiarid Mediterranean climate. Traditionally, agriculture in this area was dominated by extensive cultivation of cereal crops (mainly wheat and barley) and fallows, with some olive and almond trees in the steepest areas. During the 20th century, agriculture in this area underwent several important changes, mainly due to the introduction of different irrigation schemes, including the replacement of traditionally cultivated cereals with a variety of alternative crops (basically fodder crops, i.e., alfalfa and maize, and orchards) and a substantial decrease in the area of fallow lands and field margins [19], [20]. Nowadays the study area is composed of discrete irrigated and non-irrigated areas. Drylands consist of extensively managed winter cereal crops and fallows, olive and almond trees. Irrigated areas include intensively managed crops such as alfalfa, corn and some winter cereal; and orchard production with peach, pear, apple, nectarine and other fruit trees.

As in other regions in Europe, agricultural intensification in this area has led to decreases in biodiversity and in the breeding population size of several species [3], [6], [21]. Of particular concern has been the decline of steppe bird populations in the study area over recent decades, as it is part of the western European stronghold of many of these species [22]. In the present study we used data on ecological requirements and field censuses of four ground-nesting steppe bird species, which are still widely distributed in the study area, to implement and validate our models. These include Little Bustard *Tetrax tetrax*, Stone Curlew *Burhinus oedicnemus* and Calandra Lark *Melanocorypha calandra*, all of conservation concern and protected at European, national or regional levels, and Red-legged Partridge *Alectoris rufa*, a widespread but declining farmland game bird species of significant importance to the local rural economy.

### Bird Occurrence Data

We established a total of 145 linear transects in the study area in 2010 and 2011 (83 surveyed in both years, 40 just in 2010 and 22 just in 2011) (Fig. 1b), along which observers walked and recorded all birds either acoustically or visually detected. Transect lines had a length of ca. 500 m and were spaced more than 1 km apart. We established maximal 100 m-wide belts on each side of the transect line. Censuses were performed in May, to match observation effort to periods of high activity for three of the studied species in the study area. In contrast, detectability of red-legged partridges maybe somewhat decrease in May as compared to earlier months. However, as we used presence/absence rather than abundance data for analyses (see below), we do not think this represents a significant bias in our data. Censuses were performed from 6 a.m. to noon and only in periods with good weather conditions (with no rain and no or light wind). Transects were chosen to contain different proportions of the six dominant herbaceous cover types, i.e. combinations of crop types and cropping management for those crops, present in our study area [23]. These cover types included dry extensively-managed cereal, irrigated intensively-managed cereal, till fallow (arable land that was not cultivated for one or more seasons, and which was ploughed to avoid weed development regularly), no-till fallow (arable land that was not cultivated for one or more seasons, and where weeds were managed, if necessary, using herbicides), irrigated intensively-managed alfalfa and irrigated intensively-managed maize. These cover types together represented ≥80% of the total surface of all monitored transects. Other cover types (i.e., shrub, urban areas and orchards) were considered unsuitable as the study species rarely use them. The relative proportions of different cover types in each transect remained constant throughout the breeding period.

For the analyses below, we classified each of our four study species as either present or absent on each transect survey. We used presence/absence data rather than abundance data because of its high performance for low density or cryptic species [24], such as those monitored in the present study. Since all cover types considered are herbaceous, with broadly similar vegetation structure, and because censuses were conducted in periods of high activity of birds by either visual or acoustic contact, we do not think detectability differences among habitat types significantly biased our presence/absence data. For Little Bustards, only information on males was used for analyses, due to poor female detectability using transect line methods [25].

### Resource-based Model

**Figure 2:**
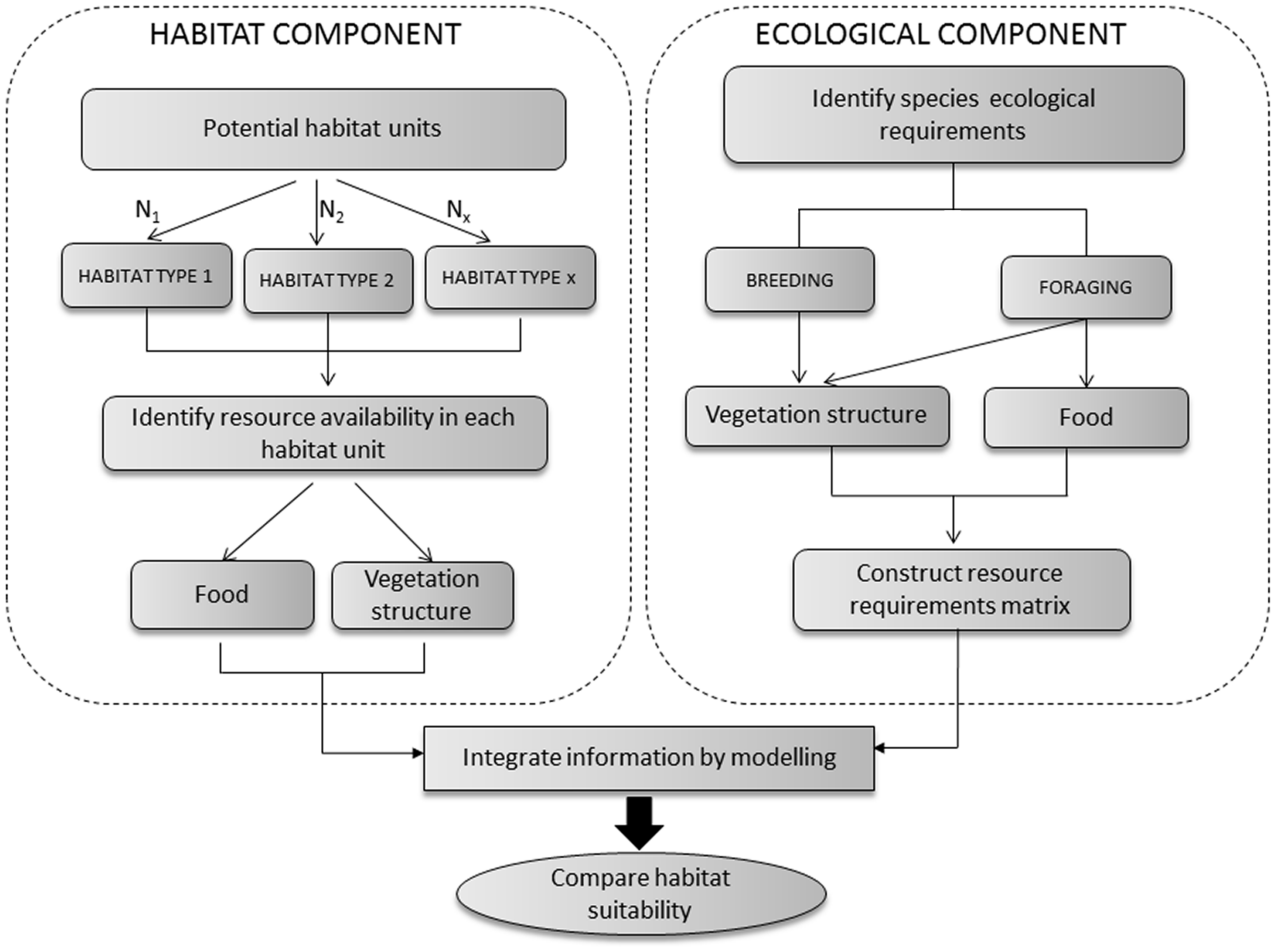
Overview of the framework followed to model the effect of different habitat types on habitat suitability for steppe bird species that was applied in this study.

Figure 2 sets out the general framework we used to model habitat suitability as a function of nesting and foraging resource availability. This approach first identifies the pool of potential habitat types in a particular study area according to their agronomic, environmental and socio-economic characteristics (in our study case and for validation purposes, these were the six dominant herbaceous cover types defined above). The framework then follows four steps: (1) the construction of a matrix to describe species’ resource requirements for each vital activity (i.e. nesting and foraging) and for each time period categorized; (2) the quantification of resource availability in each habitat type and for each time period; (3) the calculation of habitat suitability indices for each of the vital activities for each species in each habitat type and for each time period; (4) the temporal and/or spatial integration of habitat suitability indices to encompass temporal and spatial variation at the scales at which considered vital activities occur. Note that the most appropriate duration of each time period categorized will depend on both the temporal dynamics of the system being considered and species’ resource requirements. In the following subsections we give details on these steps for our case study system (a detailed example can also be found in Appendix S1).

#### Step 1: Matrix of resource requirements

Resource requirement data for each of the ecological requirements considered (i.e. foraging and nesting habitat characteristics - specifically vegetation height [26], [27] - and diet content) are described in a *species×resource* table **R = [** *r_ij_* **]** for each time period. Using data from 26 studies across the species’ main distribution range areas (Table 1), here we categorized resource requirements on the basis of preferred vegetation height for foraging or nesting, or presence of a given food resource in the diet. This allowed us to categorise resource requirements using a simple and comparable approach between species and regardless of data quality in the original publications. Four vegetation height categories (0–25 cm, 25–50 cm, 50–100 cm, >100 cm) were defined to describe foraging and nesting resources related to habitat characteristics. For each vegetation height category, the *r_ij_* value assigned reflected an assessment of the capability of species *i* for using vegetation height *j* (0– not preferred i.e. vegetation heights used only occasionally or avoided; 1– preferred i.e. vegetation heights where an important and high proportion of the individuals forage or nest). For dietary resources we considered four main food types (seeds, plants, invertebrates and vertebrates), with each *r_ij_* value reflecting an ordinal measure of the degree of preference for each food type *j* by species *i* (0 - not used i.e. food never or very rarely consumed; 0.5 - rarely used i.e. food consumed secondarily, when usual food is not widely available; 1 - preferentially used i.e. a primary and frequent food resource for a given species) [28]. Further, we evaluated the sensitivity of our resource-based model to preference values by considering three alternative ways of scoring preferences: (i) 0/0/1; ii) 0/0.25/1 and (iii) 0/0.75/1 for resources not used, rarely used and preferentially used, respectively. Values were derived for two periods, spring (April-June) and summer (July-September), to reflect temporal changes in resource requirements through the breeding season.

**Table 1:** Resource requirements of the four study steppe bird species.

	Vegetation height (cm)				Diet			
	0–25	25–50	50–100	>100	Seeds	Plants	Invertebrates	Vertebrates
**Nesting**								
Little bustard								
Calandra Lark ^[28]^	*Yes*	*Yes*	*No*	*No*				
Stone Curlew ^[48], [49]^	*Yes*	*No*	*No*	*No*				
Red-legged Partridge ^[50]–[54]^	No	Yes	Yes	Yes				
**Foraging (spring)**								
Little bustard ^[25], [55]–[58]^	*Yes*	*Yes*	*No*	*No*	*Rare*	*Usual*	*Rare*	*Not used*
Calandra Lark ^[44], [59]–[62]^	*Yes*	*Yes*	*No*	*No*	*Usual*	*Rare*	*Usual*	*Not used*
Stone Curlew ^[48], [57], [63]^	*Yes*	*No*	*No*	*No*	*Not used*	*Not used*	*Usual*	*Rare*
Red-legged Partridge ^[57], [63]–[65]^	*Yes*	*Yes*	*No*	*No*	*Rare*	*Usual*	*Rare*	*Not used*
**Foraging (summer)**								
Little bustard ^[55], [56], [66]^	*Yes*	*Yes*	*No*	*No*	*Rare*	*Usual*	*Usual*	*Not used*
Calandra Lark ^[60]^	*Yes*	*No*	*No*	*No*	*Usual*	*Rare*	*Rare*	*Not used*
Stone Curlew ^[48], [67]^	*Yes*	*No*	*No*	*No*	*Not used*	*Not used*	*Usual*	*Rare*
Red-legged Partridge^[65], [68]–[70]^	*Yes*	*Yes*	*No*	*No*	*Usual*	*Usual*	*Usual*	*Not used*

#### Step 2: Matrix of resource availability

Resource availability data for both habitat and dietary resources are described in a *habitat type* × *resource* table **A = [** *a_kj_* **]** for each time period. Each *a_kj_* value is a measure of the availability of resource *j* in habitat unit *k*. In our study case, we used available information on agricultural practices applied to different cover types in our study area (i.e., sowing and harvesting dates, fertilizers used, irrigation and plough; [23]) in combination with authors’ expert knowledge based on 10 years of field surveys, to qualitatively describe the probability of a given cover type *k* having a given vegetation height *j* (i.e., 0–25 cm, 25–50 cm, 50–100 cm, >100 cm) in each time period (0 - not possible i.e. vegetation height category never or very rarely present; 0.5 - rare i.e. infrequent or marginal vegetation height category; 1 - usual i.e. dominant vegetation height category). We then transformed these values to relative frequencies by dividing the score of each category by the sum of scores of all categories in a given period and land cover type, so that the sum of all categories was 1. We also evaluated the sensitivity of our resource-based model to the choice of probability scores by applying the following alternative scoring options for the three probability classes (not possible, rare, usual): (i) 0/0/1; (ii) 0/0.25/1 and (iii) 0/0.75/1. Values were derived monthly according to vegetation growth patterns and land management (for more detailed information, see Fig. S1).

For dietary resources, *a_kj_* values indicate the relative abundance of resource *j* in cover type *k* in a given time period. We assumed that the abundance of dietary resource *j* in cover type *k* was inversely related to both the number of agricultural practices that negatively affect that resource (*n*) and the intensity of these practices (*f*) [7], [8]. Specifically, we calculated relative food abundance for each resource as,
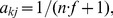
(1)where *n* · *f* +1 was used in order to avoid infinite *a_kj_* values. For these calculations, we considered the effect of three main practices known to be directly related to food abundance: agro-chemical use, irrigation and ploughing [29]. Thus, *n* values were bounded by 0 and 3. These practices can lead to reduction in food supply of our study species directly (e.g. reduction in weed availability through the use of herbicides) or indirectly (e.g. elimination through competition of many broad-leaved plant species and invertebrates associated with them by stimulation of crop growth through crop irrigation or fertilizer use) [4], [29]. We used field yield (tonnes/ha) as the scaling factor for production system intensity (*f*), with *f* for fallow lands set to 1. We also examined the sensitivity of our model to the choice of this scaling factor by: (1) setting the scaling factor to one for all agricultural systems considered; and (2) using a coarser qualitative measures describing the degree of intensification of practices (1 - low-intensive, applied to dry extensively-managed cereal, till fallow and no-till fallow; 2 - high-intensive, applied to irrigated intensively-managed cereal, alfalfa and maize). Expected food abundance was calculated for spring (April-June) and summer (July-September), based on land management (Table 2).

**Table 2:** Main agricultural practices likely to influence studied steppe bird food resources (seeds, plants, invertebrates and vertebrates) in different farming systems within the study area throughout the breeding season (spring: Sp and summer: Su).

		Unmanag.Fallow		Manag.fallow		Drycereal		Irrigatedcereal		Alfalfa*		Maize	
Practice	Key impacts	Sp	Su	Sp	Su	Sp	Su	Sp	Su	Sp	Su	Sp	Su
Agro-chemical inputs	Loss of cropplant material	+	–	–	–	+	+	+	+	+	+	+	+
	Loss of seeds	+	–	–	–	+	+	+	+	+	+	+	+
	Loss of cropinvertebrates	+	–	–	–	+	+	+	+	+	+	+	+
Irrigation	Loss of cropplant material	–	–	–	–	–	–	+	–	+	+	+	+
	Loss of cropinvertebrates	–	–	–	–	–	–	+	–	+	+	+	+
Plough	Loss of crop invertebrates	–	–	+	+	–	+	–	+	–	–	–	–
	Loss of cropplant material	–	–	+	+	–	+	–	+	–	–	–	–
	Loss of seeds	–	–	+	+	–	+	–	+	–	–	–	–
	Loss of cropvertebrates	–	–	+	+	–	+	–	+	–	–	–	–
Field yield (tonnes/ha)						2	2	7	7	14	14	12	12
Seed availability^1^		0.5	1.0	0.5	0.5	0.33	0.2	0.12	0.07	0.07	0.07	0.08	0.08
Plant availability^1^		0.5	1.0	0.5	0.5	0.33	0.2	0.07	0.07	1.0	1.0	0.04	0.04
Invertebrate availability^1^		0.5	1.0	0.5	0.5	0.33	0.2	0.07	0.07	0.03	0.03	0.04	0.04
Vertebrate availability^1^		1.0	1.0	0.5	0.5	1.0	0.33	1.0	0.12	1.0	1.0	1.0	1.0

Symbols −/+ indicate absence/presence of each practice and its key impacts on different food resources.

*In the case of alfalfa negative effects of agricultural practices were only considered to affect seeds, invertebrates and vertebrates, but not plant material since the crop by itself could be consumed by plant-eaters.

1 Availability was calculated as 1/(*n* • *f* +1), where *n* is the number of agricultural practices that negatively affect food resource considered in calculations and *f* the production system intensity (measured by field yield). For fallows *f* was set to 1.

#### Step 3: Calculation of habitat suitability for each vital activity

Habitat suitability for a particular species in a given habitat type and period considered is broadly defined as the degree of coincidence between species’ key ecological requirements and resource availability in that habitat type. For each ecological requirement considered (i.e., dietary resources, foraging vegetation height or nesting vegetation height) and period, we define a *habitat type* × *species* table **S = [** *s_ik_* **]**, where suitability *s_ik_* of habitat type *k* for species *i* is defined as the scalar product of the corresponding vectors of matrices **A** (availability) and **R** (requirements):
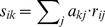
(2)


Suitability values derived from vegetation characteristics were, by definition, bounded between 0 and 1. To meet the same criterion, we truncated suitability values associated with food abundance to 1, acknowledging that species can use complementarily different food resource in relation to their availability in a given cover type and species trophic niche until total diet requirements are satisfied (but not over this threshold).

In our framework, foraging habitat suitability depends on both expected food abundance and accessibility to food due to habitat preferences (related to efficiency of foraging or predator avoidance) [17], [26]. Since both components (abundance and accessibility) are considered obligate, a multiplicative approach is used. Thus, we defined foraging suitability 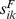 for species *i* and habitat type *k* as the product of the corresponding suitability derived from foraging habitat characteristics (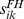) and the suitability derived from expected food abundance (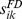):
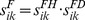
(3)


In contrast, nesting habitat suitability (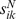) is defined as the suitability derived from nesting habitat characteristics (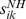) only:
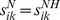
(4)


In our study case, we calculated 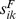 and 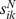 values monthly, according to the temporal resolution of vegetation height data. However, we assumed that (1) food preferences remained constant across both spring (April-June) and summer (July-September) periods and (2) expected food abundance was constant within each period but potentially varied between them. Nesting-related habitat suitability was only calculated for those months when nesting activity occurs, according to the known breeding requirements of the different species (between April and June for Calandra Lark and Red-legged Partridge and between April and July for Stone Curlew) [28]. Note that no nesting resource–related habitat suitability measures were calculated for Little Bustard, and perceived habitat suitability is therefore based only on foraging habitat for model validation; as discussed above, occurrence data for this species were based solely on displaying males, which do not take part in nesting activities, and nests do not necessarily occur within territories of displaying males [30]. All indices were implemented in R software (R Development Core Team, 2011).

#### Step 4: Integration of habitat suitability estimates

The final objective of our modelling framework is to obtain single habitat suitability estimates to represent probability of occurrence for each target species in the periods and areas of interest. Integration of habitat suitability estimates will depend on spatial and temporal variation of habitat suitability estimates and species’ ecology, as well as on the purpose of the application. For model validation in our study case, spatial integration was done at the scale of surveyed transects (200 m×500 m), which was considered to be broadly representative of the scale of habitat used by studied species during the breeding season (or territories of displaying males in the case of Little Bustard, see above). We calculated foraging and nesting habitat suitability estimates for species *i* in each monitored transect *t* (hereafter, 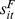 and 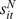) as weighted averages of 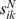 and 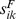 estimates across all cover types present in such transect. In these calculations we defined the weight of each cover type *k* as its relative proportion within the 200 m×500 m area encompassed by the transect belt. Relative proportions of such cover types were calculated with respect to the total transect surface, which takes into account that in some transects unsuitable habitats (i.e., shrub, urban areas and orchards) were also present (see above in bird occurrence data). In this way, we considered the effects of both the quality and quantity of available resources on final habitat suitability [15].

Next, temporal integration of monthly 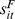 and 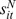 estimates was conducted by averaging monthly values across: (1) the complete breeding season (i.e. April – September); and (2) different windows of time around bird surveys: (2a) the month when the survey was undertaken (May); (2b) including the month before sampling (Apr-May); (2c) including one month after sampling (May-Jun); and (2d) including one month either side of sampling (Apr-Jun). This was done to evaluate the strength of the relationship between May occurrence data and suitability over different time periods because, although it is known that habitat suitability throughout the complete breeding season is important for species’ fitness, habitat selection patterns may respond to more discrete periods [31], [32]. For temporal integration of 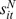, only months within nesting period were taken into account. Finally, since both nesting and foraging habitat suitability are essential to ensure population viability [33], we calculated total habitat suitability as the geometric mean of nesting-and foraging-related habitat suitability (i.e., that total habitat suitability is 0 if one of these components is 0). All analyses were implemented in R software.

### Habitat-based Model

We employed generalized linear models (GLM) to analyse factors affecting species occurrence (binomial error distribution; logit-link function), using presence/absence of each species during bird census as the dependent variable. For duplicate samples (i.e., transects monitored in both years) and to avoid pseudoreplication, one sample was randomly selected to be used in models. We used the percentage of each herbaceous cover type (i.e. dry extensively-managed cereal, irrigated intensively-managed cereal, no-till fallow, till fallow, irrigated intensively managed alfalfa, irrigated intensively-managed maize) and a category ‘other’ to account for additional cover types as the fixed effects. Both the linear and quadratic forms of these variables were tested. Using a multimodel inference approach [34], model-averaged parameter estimates were derived on the basis of corrected Akaike’s information criteria (AICc) for all possible subsets of models constructed from combinations of these variables. Multimodel inference was implemented in R software by the functions ‘dredge’ and ‘model.avg’ from the ‘MuMIm’ library.

We used a five-fold cross-validation procedure to generate model predictions, so that data used for model assessments were independent from the data used for calibration. In this way, the occurrence dataset was randomly divided into 5 independent partitions, with four used for model calibration and the remaining partition (20% of data) for model assessment. This procedure was repeated five times, so that we obtained a prediction for each considered transect.

### Model Validation and Comparison

We relied on three indices to compare model predictions to observed species’ occurrence: prediction success (i.e., proportion of correctly predicted observations), sensitivity (i.e., proportion of correctly predicted presences) and specificity (i.e., proportion of correctly predicted absences). For these analyses, we used the value of presence probability that maximized the sum of sensitivity plus specificity as a threshold to transform our model predictions to presence/absence data [35]. Further, we also used AUC (Area Under the Receiver Operating Characteristic Curve) as a threshold independent measure of model performance. Model performance was assessed using the functions ‘somers2’, ‘optim.thresh’ and ‘accuracy’ from the ‘Hmisc’ and ‘SDMTools’ libraries in R software.

### Scenario Assessment

As an example of its application, we use our resource-based model to predict the potential effects, on the study area, of removing the set-aside support across Europe following the 2008 CAP (Common Agricultural Policy) ‘Health check’, which has generated strong debate over its possible negative impacts on biodiversity conservation [36], [37]. Under this policy decision, the most likely agronomic scenario for our study area includes the loss of fallow land in favour of cereal fields [38], with consequent changes in habitat structure and food availability through the breeding season. This process was not apparent in the study area by the date of this study, but could lead to important changes in habitat composition in the near future. At the time of our surveys, the study area comprises 71% of dry cereal fields, 3% of till fallow, 7% of no-till fallow, 1% of maize, 9% of irrigated cereal and 4% of alfalfa. Here, we assessed the effect on the habitat suitability for each species of shifting 30%, 50% and 100% of current fallow land (till plus no-till) to dry cereal within the study area. We also compared resource-based model predictions with predictions obtained using the habitat-based models. For these analyses, only models considered sufficiently robust (AUC ≥0.6) were used [39]. As with the transect data, probability of occurrence was calculated as the average suitability values across habitat types, using their proportions in each agronomic scenario as weights. Further, we translated model predictions into presence/absence data at the transect scale using threshold values obtained in the model validation (see above and in Table 3). In all calculations we assumed that proportion of till and no-till fallow systems lost will be equal.

**Table 3:** Model performance of habitat-based and resource-based models for predicting the occurrence of four steppe bird species in the study area.

Species	Success(%)	Sensitivity(%)	Specificity(%)	AUC	Threshold
***Habitat-based model***					
RP	50	65	42	0.47	0.33
SC	55	77	51	0.64	0.11
CL	70	82	61	0.76	0.44
LB	70	26	85	0.52	0.34
***Resource-based model***					
RP	39(37–41)	100(96–100)	10 (10–15)	0.46(0.42–0.44)	0.46(0.27–0.39)
SC	77(68–77)	55(55–64)	80 (68–80)	0.66(0.66–0.69)	0.26(0.09–0.18)
CL	70(64–69)	82(82–85)	60 (49–55)	0.74(0.66–0.72)	0.33(0.21–0.38)
LB	72(71–73)	45(39–47)	82 (79–85)	0.65(0.59–0.65)	0.42(0.32–0.42)

For habitat-based models, model performance according to predictions obtained using cross-validation is shown. For resource-based models, model performance of resource-based suitability estimates for the whole breeding season and for different windows of time across bird surveys (in parenthesis) are shown. The threshold that maximizes the sum of sensitivity plus specificity is also given. RP = Red-legged Partridge, SC = Stone Curlew, CL = Calandra Lark, LB = male Little Bustard. N  = 145.

## Results

### Cover Types and Resourced-based Habitat Suitability Estimates

**Figure 3:**
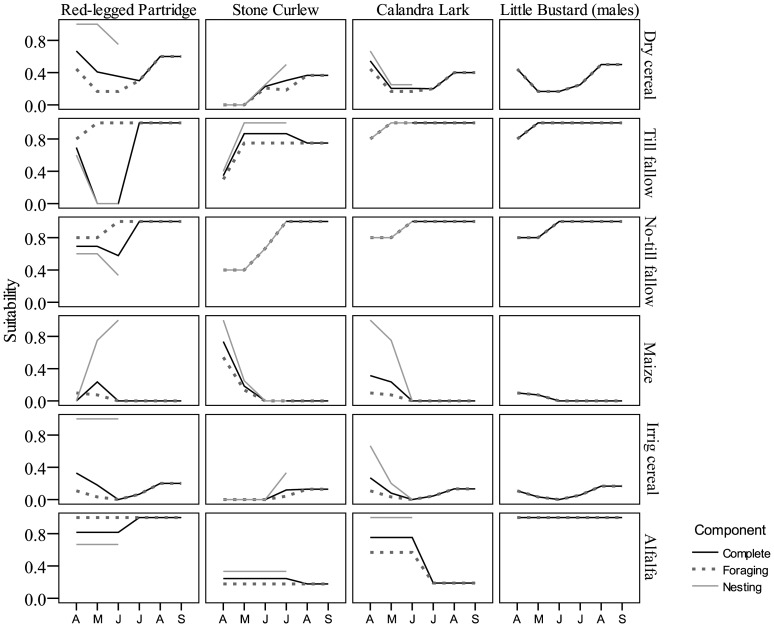
Habitat suitability estimates according to different key ecological requirements of four steppe bird species throughout the breeding season in the study area. Monthly total habitat suitability estimates were obtained as the geometric average of nesting- and foraging-related suitability estimates for months when the species is nesting and equal to foraging-related suitability estimates for months when the species is not nesting. The nesting period was bounded between April and June for Calandra Lark and Red-legged Partridge and between April and July for Stone Curlew. For male Little Bustards, no nesting period was considered since only females take part in nesting.

Estimated habitat suitability based on the resource-based models for each species varied markedly both between cover types considered and throughout the breeding cycle (Fig. 3). Overall, the highest foraging and nesting habitat suitability estimates were calculated for fallow systems (till and no-till), particularly late in the season when they offered a low vegetation height (see Fig. S1) and higher expected food abundances than the other systems (Table 2). Temporal variation of total habitat suitability estimates, was also detected at the transect scale, where monthly habitat suitability estimates were highly correlated with those expected based just on the most abundant cover type in that transect (Pearson correlation coefficients for different species and months ranges between 0.8 and 0.9). Dominant cover types represented on average 76±16% of the surface of monitored transects.

### Resource-based Model Validation and Sensitivity Analyses

The agreement between observed and predicted occurrence was reasonable for Stone Curlew, Calandra Lark and Little Bustard (AUC range: 0.65–0.74); resource-based models correctly classified between 70 and 77% of total observations for these three species (Table 3). For male Little Bustard and Stone Curlew, specificity values were superior than sensitivity values, suggesting that a considerable proportion of presences occur at locations with low habitat suitability. On the contrary, sensitivity values were larger than specificity for Calandra Lark. Prediction success was low for Red-legged Partridge (AUC <0.6). Model performance using habitat suitability calculated for different sub-periods of the breeding cycle was highly in accordance with habitat suitability calculated for the whole breeding season (Table 3) and no clear pattern of variation was observed. Sensitivity analyses for model parameters resulted overall in model presence/absence predictions being only slightly affected by alternative parameter values (≤10% of change in habitat suitability predictions at the transect level) for Stone Curlew and Calandra Lark, although changes in the particular value of habitat suitability could be higher (Table 4). However, presence/absence prediction variation was high for Little Bustard in relation to scaling factor used for production system intensity, and for the Red-legged Partridge in relation to scores for food preferences and habitat availability (Table 4).

**Table 4:** Sensitivity analyses showing the percentage of variation in the mean habitat suitability value (index), Pearson’s correlation coefficient (r) and the percentage of presence/occurrence predictions that change (prediction) at the scale of monitored transects according to changes in vegetation height, diet preferences and degree of intensification scores with respect to original values.

Parameter combinations	Red-legged Partridge			Stone Curlew			Calandra Lark			Little Bustard (male)		
	Index	r	Prediction	Index	r	Prediction	Index	r	Prediction	Index	r	Prediction
*Vegetation height*												
0/0/1	−5.4	79	21	−9.1	99	1	55.8	99	1	−3.8	97	3
0/0.25/1	−1.9	81	19	−3.4	97	3	4.5	92	8	−1.5	98	2
0/0.75/1	1.2	95	5	2.3	99	1	−3.2	94	6	1.1	99	1
*Diet preferences*												
0/0/1	55.8	95	5	65.9	99	1	62.6	97	3	83.4	92	8
0/0.25/1	4.5	99	1	10.9	99	1	7.7	99	1	13.7	98	2
0/0.75/1	−3.2	90	10	−7.9	95	5	−6.5	93	7	−10.0	98	2
*Intensification*												
Scaled	−21.9	95	5	−15.6	95	5	−16.3	94	6	−40.2	59	41
Coarsely scaled	−26.4	88	12	−18.7	95	5	−18.7	94	6	−43.9	45	55

Positive and negative values for changes in habitat suitability index indicate the direction of the change (increase vs. decrease).

### Habitat-based Models and Model Comparison

The agreement between observed and predicted occurrence, based on multimodel inference, was again reasonable for Stone Curlew and Calandra Lark (AUC was 0.64 and 0.76, respectively). Indeed, all measures of model performance indicated that habitat-based models performed very similarly to resource-based models for these species (Table 3), with strong correlation in transect-level predictions between the two approaches (Calandra Lark: r  = 0.60; Stone Curlew: r  = 0.63). In contrast, prediction success for Little Bustard and Red-legged Partridge was low (AUC  = 0.52 and 0.47 respectively) and correlations between predictions were weaker (male Little Bustard: r  = 0.53; Red-legged Partridge: r  = 0.20).

### Scenario Assessment

**Figure 4:**
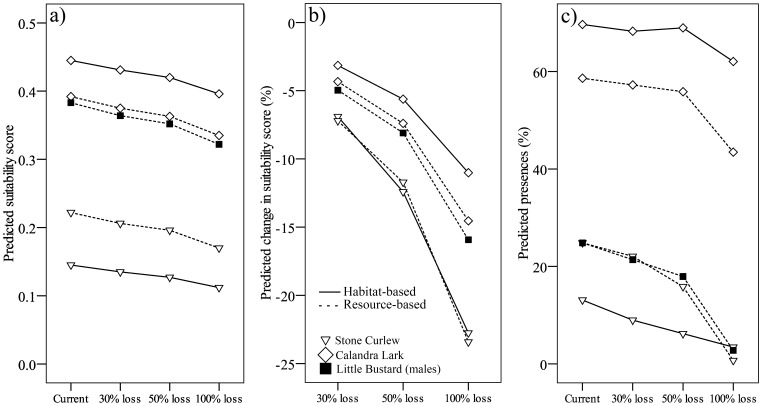
Habitat suitability for Stone Curlew, Calandra Lark and male Little Bustard in the study area derived from current landscapes and from the loss of 30%, 50% and 100% of current fallow land in the study area. Predicted a) habitat suitability, b) percentage of habitat suitability change and c) proportion of transects predicted as presences. Predictions from sufficiently robust (AUC >0.6) habitat-based models and resource-based models are shown for each species and scenario.

**Figure 5:**
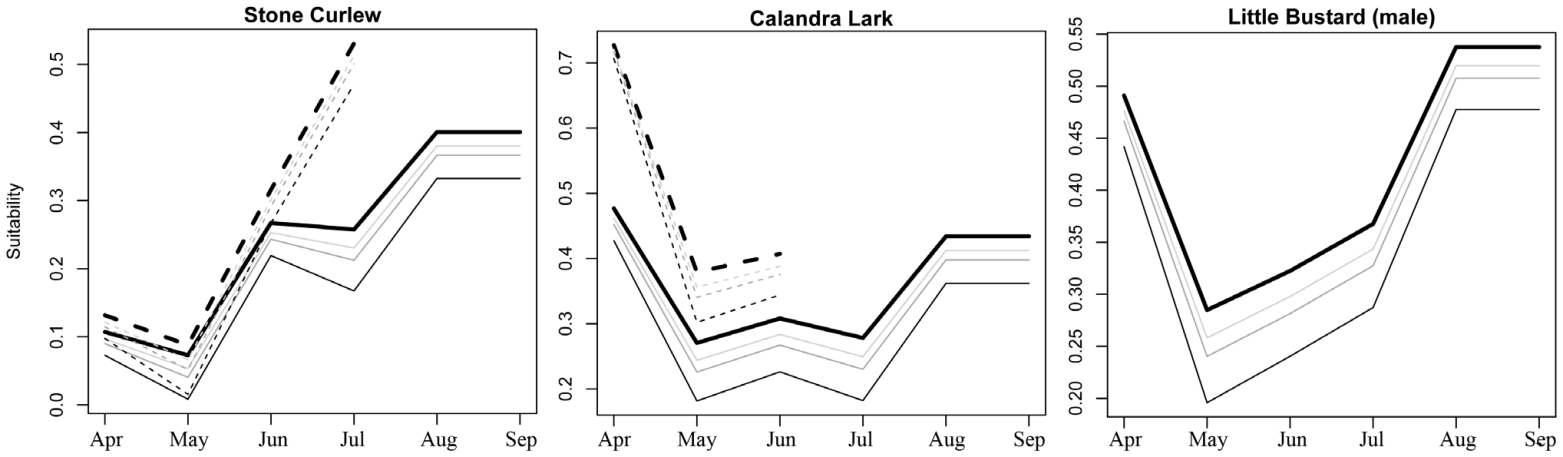
Temporal variation of predicted habitat suitability according to different key ecological requirements for Stone Curlew, Calandra Lark and male Little Bustard in the study area derived from current landscapes (thick black line) and from the loss of 30%, 50% and 100% of current fallow land in the study area (thin light grey, dark grey and black lines, respectively). Solid lines indicate foraging-related habitat suitability, while dashed lines indicate nesting-related habitat suitability.

Habitat suitability for male Little Bustard, Calandra Lark and Stone Curlew was predicted to decrease between 4% and 23% in the study area as a result of a 30% to 100% decrease in the proportion of fallow land (Fig. 4a,b). These trends were very similar to those produced by habitat-based models in the study area for Stone Curlew and Calandra Lark (Fig. 4c). Expected changes in habitat suitability values were not equivalent for all months of the breeding season according to the resource-based models (Fig. 5). This was particularly apparent in the case of Calandra Lark and Little Bustard, for which major losses of foraging- and nesting-habitat suitability (for Calandra Lark) were expected to occur between May and July, which are also the months of the breeding season with lowest suitability values.

## Discussion

In this study, we developed a resource-based model, whereby land uses and agricultural practices are defined in terms of availability of key foraging and nesting resources for target species [7], [8]. Habitat suitability estimates generated by our models were congruent to independent species’ occurrence data in our study area and overall performed similarly (and better in the case of one study species) to habitat-based models based on current distributions. Acceptable resource-based models were obtained for three out of four species considered (AUC: 0.65–0.74), suggesting that overall the assumptions of the model structure are reasonable for these species, but at the limit of what can be considered of useful application according to standards posed by several authors (i.e., AUC  = 0.7). However, given that these results arose from application of our models to relatively homogeneous extensive agricultural landscapes (the most suitable region for these species in Catalonia, most of it included in the Natura 2000 network) and that we use presence/absence data for model validation, which tends to be insensitive to small variations in habitat suitability, we take them as support and encouragement for further work to improve our resource-based models in the near future.

Contrary to the other species studied, our resource-based suitability estimates failed to predict the occurrence of Red-legged Partridge in the study area. This could be due to a number of factors. First, our resource-based model might be conservative since it assumes that if suitable habitat exists, species will be able to access and use it. However, this might not be the case if populations are maintained at low-density (for example because they are hunted), or there are other abiotic or biotic constraints (e.g. microclimate, topography or interspecific interactions) that also limit current ranges. The former process might be occurring to the Red-legged Partridge in the study area, which is subject to high hunting pressure [22]. In this situation it might be expected that resource-based models over-predict species current range. Consistent with this prediction, the sensitivity index (percentage of correctly classified presences) clearly exceeded the specificity index (percentage of correctly classified absences) for this species. The fact that censuses were conducted in May, which is relatively late in the breeding season for this species, might also contribute to such result. Furthermore, compared to other study species, the Red-legged Partridge may be considered as a habitat generalist [28]. It may be that our inability to adequately predict occurrence of this species (by both resource-based and habitat-based models) is underpinned by a more general pattern related to the difficulty of estimating resource requirements in generalist species due to inter-individual variability [10], [40]. Currently this limitation is an unsolved problem in distribution modelling and might affect both resource-based and habitat-based models [10].

For Stone Curlew and Calandra Lark, resource-based and habitat-based models performed similarly when predicting current species local distribution but the former performed better for Little Bustard. This relatively small improvement in predictive capability associated with the resource-based models, despite the additional time and data required for their implementation, may be related to the fact that, although not explicitly considered, resource-provisioning might be implicitly incorporated in habitat-based models when applied to current conditions. For example, if a strong linear relationship between the area of a particular cover type and the availability of the key resource type underpinning a species’ habitat selection exists, one would expect to see a strong association between the species and that habitat type [11]. However, these relationships are often context specific (both temporally and spatially) and particular anthropogenic definitions of cover types may not necessarily closely reflect the same underlying resource availability in times or places other than that of model calibration, thus limiting the effectiveness of conservation strategies based on these approaches [15], [41]. Greater differences in predictive capability would therefore be expected if, for example, the models were used to predict occurrence in other areas or the impacts of changes such as altered management practices that are not easily incorporated into habitat-based models.

By allowing the explicit incorporation of management practices, as defined by farmers/agronomists, into functional habitat types, resource-based models may also enhance the establishment of links between the languages of conservationists and farmers/agronomists, who are ultimately responsible for the management of agricultural systems and the implementation of the majority of conservation measures developed [42]. For example, while wide consensus exists among conservationists about the positive role of fallow land on farmland biodiversity [37], [43], [44], it is rarely taken into account that, according to farmer/agronomist management, different types of fallows may exists (e.g. till or no-till fallow in our study area) and that they may be perceived as different habitats by bird species.

As with habitat-based models, resource-based models are limited by the availability of appropriate data and also the mechanistic links between ecological requirements and resource provisioning need to be sufficiently understood. For example, in our study we used vegetation height as a measure of foraging habitat and nesting resources because we were working with ground-nesting farmland birds, for which vegetation height has been described as a good measure of nesting and foraging habitat but, for example, information on the type of forest and its horizontal and vertical structure might be required to if using this approach to quantify habitat suitability for forest birds [45].

In this regard, the development of our resource-based models required a detailed revision of available information on farming systems and steppe birds, a process that also highlighted key data limitations and areas requiring further research. Firstly, our spatial-temporal integration of habitat suitability along monitored transects assumes that species’ occurrence depends on the crop surface along those transects (500 m×200 m). Since this level of spatial resolution is broadly representative of the scale of habitat used during the breeding season by our study species, we do not think that this assumption introduced significant bias into our results. However, including finer resolution information of the scale at which nesting and foraging habitat use occur would likely improve model accuracy [41], [46]. Secondly, our assessment of habitat suitability is based solely on the structure and management of the cropped areas during spring and summer. However resource availability over winter can also limit bird distribution and a model incorporating habitat suitability for the whole year would thus likely improve model accuracy for sedentary species [15]. Additionally, due to the scarcity of quantitative information on food abundance and vegetation structure in the different agricultural systems present in our study area, expected food abundance and vegetation structure had to be calculated qualitatively and at the cover-type level according to their different management regimes. Whilst we believe this assumption is justified for the analyses presented here, based on previous information of the effect of considered agricultural practices and their intensity on resource provisioning [29], further research to quantitatively identify food abundance and vegetation structure in different agricultural systems, as well as intra-system variations over the breeding season would allow more accurate predictions to be made. In a similar way, while using qualitative information on species’ resource requirements allowed direct comparisons between species for which the amount and quality of information is highly variable, iterative refinement of model parameters (through collection of more experimental or observational data or model calibration to other existing distribution data sets) would also allow more accurate predictions to be made [11], [17]. In this respect, sensitivity analyses can help to clarify the proportion of the total error that might be accounted for by uncertainty in models parameters, while also providing valuable insights for prioritizing new data collection [10]. For example, our sensitivity analyses suggested that, while availability and preferences for different vegetation structure and food resources are important, collecting empirical data on management intensity is a higher priority, at least for Little Bustard. Further, our models indicate that habitat suitability explained the probability of occurrence (and thus distribution), but it would be worthwhile in future works exploring the relationship between suitability and abundance as this would provide greater resolution to our understanding of species’ responses to changes in resource availability [47]; predicting and responding to population decline may also be more efficient and effective than predicting and responding to changes in species’ distribution. Finally, predictive abilities of our resource-based models, which were based on vegetation height and food requirements, may be improved by considering additional requirements, such as vegetation cover or heterogeneity, or modulating the suitability of habitats in relation to distance to unsuitable areas. Further work may explore the convenience of the inclusion of such type of additional information for model improvements.

## Conclusions

Frameworks for assessing the potential effects of different land-use alternatives on biodiversity are fundamental for guiding objective management decisions. Resource-based models, such as the one developed here, provide a structure for using an understanding of the functional links between agricultural practices, provision of key resources and the response of organisms, to predict potential effects of changing land-uses on habitat suitability. When management alternatives lie within the range of agricultural options available in a given area and management practices are expected to remain constant, resource-based and habitat-based models seem to offer overall similar predictions. However, if new cover types or new management strategies are introduced (for which habitat-based models are not parameterised), resource-based models offer a structure for integrating inter-disciplinary knowledge (agronomic and ecological knowledge) to allow the impact of those changes to be evaluated. These models could be iteratively refined (through collection of more experimental or observational data or model calibration to other existing distribution data sets) so that more accurate model parameters can be incorporated. However, a key challenge is to provide models that are sufficiently accurate and general to enable application to a variety of temporal and spatial context while remaining feasible to parameterise. Conservation of threatened species in humanized landscapes has not always been addressed in a multidisciplinary and mechanistic way where, by integrating information on possible land-uses or land-management practices and species’ key ecological requirements, habitat suitability could be determined *a priori*. However, the development of frameworks that allow establishing explicit links between agronomic and environmental realities in humanized landscapes are essential to inform decision-making processes and to design agronomic solutions delivering acceptable trade-offs between agricultural production and conservation.

## Supporting Information

Figure S1 **Crop vegetation height in different agricultural systems considered in our study throughout the breeding season, according to agricultural practices applied and author’s expert knowledge.**
(DOC)

Appendix S1 **General modelling framework: an example of application.**
(DOCX)
