# Correction: A Resource-Based Modelling Framework to Assess Habitat Suitability for Steppe Birds in Semiarid Mediterranean Agricultural Systems

**DOI:** 10.1371/journal.pone.0104319

**Published:** 2014-07-28

**Authors:** The PLOS ONE Staff

There are errors in the Funding section. The correct funding information is as follows: This study was supported by the Projects “Steppeahead” funded by Fundación General del Consejo Superior de Investigaciones Científicas from Spain (FGCSIC), CSIC and Banco Santander, “TRUSTEE” funded by RURAGRI ERA-NET (No 235175) and “Farmland” funded by ERA-NET BIODIVERSA (PRI-PIMBDV-2011-0880). F.C. was supported by a JAE-Doc contract financed by CSIC and the European Social Fund (ESF). L.C. was supported by a postdoctoral contract funded by FGCSIC and Banco Santander. FARMDINDIS project, funded by Infraestructures.cat (Generalitat de Catalunya) and Aigües del Segarra-Garrigues SA, provided data on field censuses. The funders had no role in study design, data collection and analysis, decision to publish, or preparation of the manuscript.
