# Supplementary material for: A Resource-Based Modelling Framework to Assess Habitat Suitability for Steppe Birds in Semiarid Mediterranean Agricultural Systems
Source: PLoS One. 2014 Mar 25;9(3):e92790. doi: 10.1371/journal.pone.0092790 (PMC3965467; doi:10.1371/journal.pone.0092790)

**Figure S1.** Crop vegetation height in different agricultural systems considered in our study throughout the breeding season, according to agricultural practices applied and author’s expert knowledge. Relative frequencies of different vegetation height categories (0-25 cm in white, 25-50 cm in light grey, 50-100 cm in dark grey, >100 cm in black) obtained by dividing scores assigned to each vegetation category according to its probability of occurrence (0 not possible – vegetation height categories never or very rarely present -, 0.5 rare – infrequent or marginal vegetation height categories -, and 1 usual – dominant vegetation height categories) by the sum of scores of all categories in a given period are shown. For graphical purposes categorical estimates of vegetation heights are also presented as continuous weighted mean height values (in red), calculated as the frequency of crops assigned to each height categories in each agricultural system multiplied by the mid-point of each height class (i.e., 12.5, 37.5, 75 and 100m respectively). For cereal fields, which are sown in November, increased proportion of height fields are expected throughout the breeding season, until harvesting dates (between 1 jun – 31 jul for dry cereal and 15 jun – 31 jul for irrigated cereal). Once harvested, cereal fields are plough in the study area and so, no stubbles are present. Alfalfa fields which are regularly harvested during the breeding season, presented low vegetation heights during all the season. Maize fields are sown in April and presented increased heights throughout the season. Till fallows, which are plough in early spring and early summer, presented low vegetation heights during major part of the breeding season. No-till fallows, are expected to have low vegetation eights in summer because herbicides were applied in late spring.


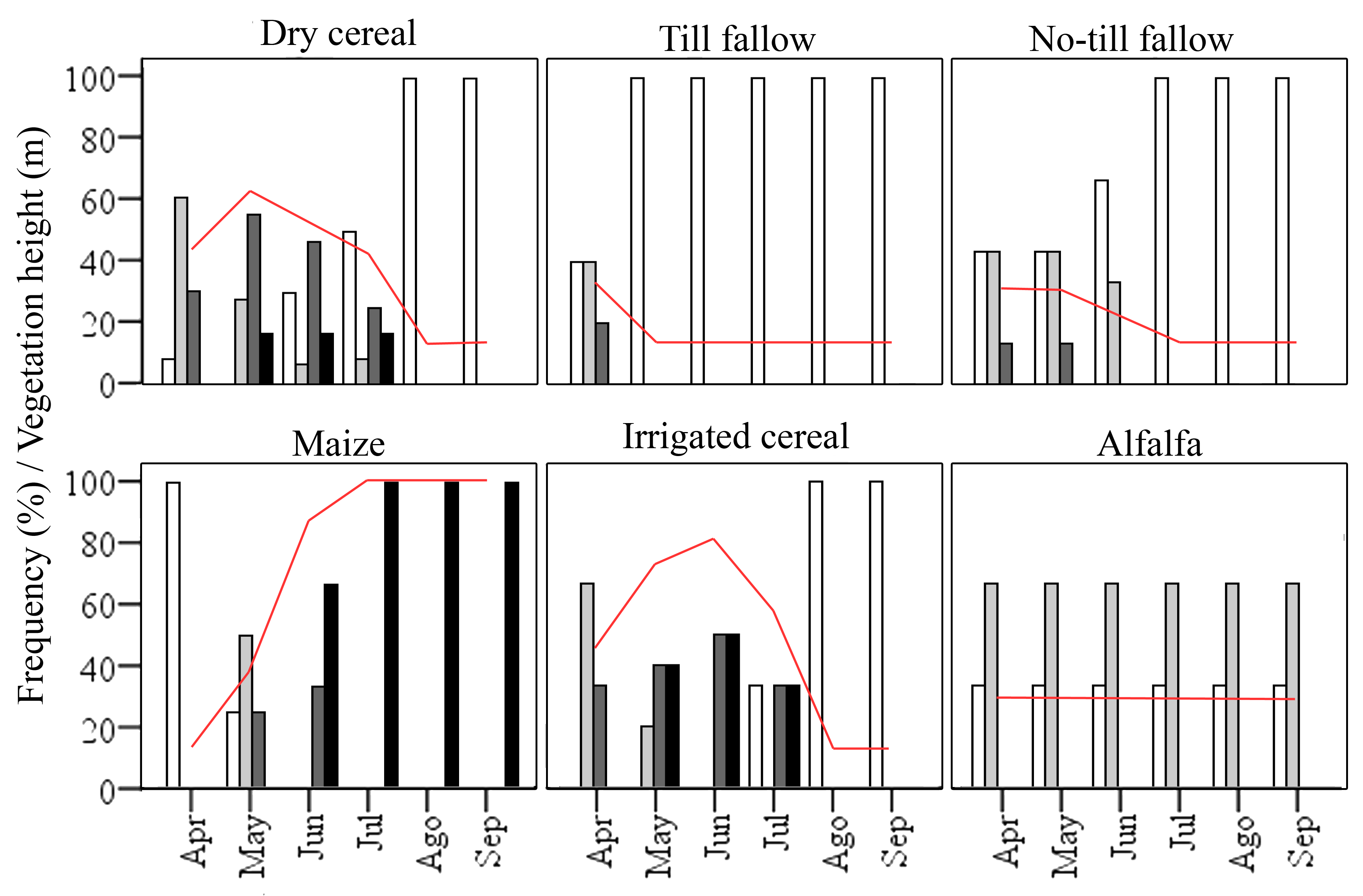

Supplement: Figure S1 — Crop vegetation height in different agricultural systems considered in our study throughout the breeding season, according to agricultural practices applied and author’s expert knowledge. (DOC) [file pone.0092790.s001.doc]
