# Supplementary material for: A Resource-Based Modelling Framework to Assess Habitat Suitability for Steppe Birds in Semiarid Mediterranean Agricultural Systems
Source: PLoS One. 2014 Mar 25;9(3):e92790. doi: 10.1371/journal.pone.0092790 (PMC3965467; doi:10.1371/journal.pone.0092790)
Supplement: Appendix S1 — General modelling framework: an example of application. (DOCX) [file pone.0092790.s002.docx]

**Appendix S1. General modeling framework: an example of application**

**STEP 1.** We built resource requirement matrices for the studied species by gathering data on their dietary, foraging habitat and nesting habitat requirements throughout the breeding season. Resource requirement data for each species was recorded in the form of a *species × resource* table **R** = **[***r_ij_***]** for each of the time periods to be distinguished. Four habitat types were defined according to vegetation height (0-25 cm, 25-50 cm, 50-100 cm, >100 cm) to describe foraging and nesting resources related to habitat characteristics. For vegetation heights, each *r_ij_* was an assessment of the capability of species *i* for using vegetation height *j* (0 not usable, 1 usable). In the example below, registered information for Red-legged Partridge *Alectoris rufa* (RP) and Calandra Lark *Melanocorypha calandra* (CL) is shown. Values were derived for two periods for foraging, spring (April-June) and summer (July-September), to reflect differences in resource requirements through the breeding season and only for spring for nesting, according to the species phenology.

|  | |  |  | Vegetation height (m) | | | |
| --- | --- | --- | --- | --- | --- | --- | --- |
| Species | Vital activity | | Period | 0-25 | 25-50 | 50-100 | >100 |
| RP | Nesting | | Spring (Apr-Jun) | 0 | 1 | 1 | 1 |
| RP | Foraging | | Spring (Apr-Jun) | 1 | 1 | 0 | 0 |
| RP | Foraging | | Summer (Jul-Sept) | 1 | 1 | 0 | 0 |
| CL | Nesting | | Spring (Apr-Jun) | 1 | 1 | 0 | 0 |
| CL | Foraging | | Spring (Apr-Jun) | 1 | 1 | 0 | 0 |
| CL | Foraging | | Summer (Jul-Sept) | 1 | 0 | 0 | 0 |

For dietary resources we considered three main food types, seeds, plants and invertebrates, with each *r_ij_* value being an ordinal measure of the degree of preference for each food type *j* by species *i* (0 not used, 0.5 rarely used, 1 preferentially used). See example for Red-legged Partridge and Calandra Lark below.

| Species | Period | Seeds | Weeds | Invertebrates | Vertebrates |
| --- | --- | --- | --- | --- | --- |
| RP | Spring (Apr-Jun) | 0.5 | 1 | 0.5 | 0 |
| RP | Summer (Jul-Sept) | 1 | 1 | 1 | 0 |
| CL | Spring (Apr-Jun) | 1 | 0.5 | 1 | 0 |
| CL | Summer (Jul-Sept) | 1 | 0.5 | 0.5 | 0 |

**STEP 2.** We characterized resource availability (i.e. vegetation height and food supply) through the breeding season in different habitat units. Resource availability data needs to be available in the form of a *habitat unit* × *resource* table **A** **= [***a_kj_***]** for each time period to be distinguished. For foraging and nesting habitat resources, a given *a_kj_* value indicates the relative frequency of vegetation height category *j* in agricultural production system *k* for a given time period. These values were calculated monthly, according to vegetation growth patterns and land management. In the example below, vegetation height availability in dry cereal is shown.

|  |  | | Vegetation height (m) | | | |
| --- | --- | --- | --- | --- | --- | --- |
| Production system | | Period | 0-25 | 25-50 | 50-100 | >100 |
| Dry cereal | | April | 0.00 | 0.67 | 0.33 | 0.00 |
| Dry cereal | | May | 0.00 | 0.25 | 0.50 | 0.25 |
| Dry cereal | | June | 0.25 | 0.00 | 0.50 | 0.25 |
| Dry cereal | | July | 0.50 | 0.00 | 0.25 | 0.25 |
| Dry cereal | | August | 1.00 | 0.00 | 0.00 | 0.00 |
| Dry cereal | | September | 1.00 | 0.00 | 0.00 | 0.00 |

For dietary resources, *a_kj_* values indicate the expected abundance of resource *j* in agricultural production system *k* in a given time period. Expected food abundance could be calculated for spring (April-June) and summer (July-September), based on land management. Below, expected food supply in dry cereal is shown.

| Production system | Period | Seeds | Weeds | Invertebrates | Vertebrates |
| --- | --- | --- | --- | --- | --- |
| Dry cereal | Spring (Apr-Jun) | 0.33 | 0.33 | 0.33 | 1 |
| Dry cereal | Summer (Jul-Sept) | 0.2 | 0.2 | 0.2 | 0.33 |

**STEP 3.** For each vital activity and period we define a *habitat unit* × *species* table **S** **= [***s_ik_***]**, where suitability *s_ik_* of habitat unit *k* for species *i* is defined as the scalar product of the corresponding vectors of matrices **A** (availability) and **R** (requirements) for the resource type and the vital activity considered (e.g., food types and habitat characteristics for foraging or habitat characteristics for nesting):


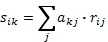


In the example below monthly suitability values for nesting and foraging vegetation height for Red-legged Partridge and Calandra Lark in dry cereal lands is shown. For these calculations, we assumed that vegetation height and food preferences and food supply were constant throughout spring (April-June) and summer (July – September).

| Vital activity | Period | Suitability index for vegetation height | |
| --- | --- | --- | --- |
|  |  | RP | CL |
| Nesting | April | 1 | 0.67 |
| Nesting | May | 1 | 0.25 |
| Nesting | June | 0.75 | 0.25 |
| Foraging | April | 0.67 | 0.67 |
| Foraging | May | 0.25 | 0.25 |
| Foraging | June | 0.25 | 0.25 |
| Foraging | July | 0.50 | 0.50 |
| Foraging | August | 1.00 | 1.00 |
| Foraging | September | 1.00 | 1.00 |

Results for suitability index for food supply was as follows,

| Period | Suitability index for food supply | |
| --- | --- | --- |
|  | RP | CL |
| April | 0.67 | 0.83 |
| May | 0.67 | 0.83 |
| June | 0.67 | 0.83 |
| July | 0.60 | 0.40 |
| August | 0.60 | 0.40 |
| September | 0.60 | 0.40 |

Finally, in our framework, foraging habitat suitability depends on both habitat characteristics and availability of food resources. Specifically, we define foraging suitability $s_{ik}^{F}$for species *i* and habitat unit *k* as the product of the corresponding suitability derived from foraging habitat characteristics ($s_{ik}^{FH}$) and the suitability derived from food availability ($s_{ik}^{FD}$):

$$s_{ik}^{F}=s_{ik}^{FH}\cdot s_{ik}^{FD}$$

In contrast, nesting habitat suitability ($s_{ik}^{N}$) is defined as the suitability derived from nesting habitat characteristics ($s_{ik}^{NH}$) only:

$$s_{ik}^{N}=s_{ik}^{NH}$$

The application of these formulations results in the following nesting- and foraging-related habitat suitability estimates for Red-legged Partridge and Calandra Lark in dry cereal systems in the study area throughout the breeding cycle. Nesting period was bounded between April and May for both Red-legged Partridge and Calandra Lark.

| Period | Nesting suitability | | Foraging suitability | | |
| --- | --- | --- | --- | --- | --- |
|  | RP | CL | RP | CL | |
| April | 1 | 0.67 | 0.45 | 0.56 | |
| May | 1 | 0.25 | 0.17 | | 0.21 |
| June | 0.75 | 0.25 | 0.17 | 0.21 | |
| July | - | - | 0.30 | 0.20 | |
| August | - | - | 0.60 | 0.40 | |
| September | - | - | 0.60 | 0.40 | |
